# Salvage therapy for relapsed testicular cancer

**DOI:** 10.18632/oncotarget.20573

**Published:** 2017-08-25

**Authors:** Nabil Adra, Lawrence H. Einhorn

**Keywords:** testicular cancer, germ cell tumor, high-dose chemotherapy, survival, prognosis

Substantial advances have been made in the treatment of testicular cancer and these have been among the great achievements in modern medicine. The introduction and refinement of cisplatin-based combination chemotherapy revolutionized the treatment of germ-cell tumors (GCT) and patients who once had a dismal prognosis are now curable even in the presence of metastatic disease [1]. Despite these advances, 20% of patients receiving first-line chemotherapy require some form of salvage therapy. Patients who relapse with anatomically confined disease can be cured by salvage surgery [2]. The vast majority of relapsed patients, however, will have disseminated disease and are treated with salvage standard-dose or high-dose chemotherapy (HDCT). Standard-dose salvage chemotherapy consists of cisplatin-based regimens with drugs not previously used: etoposide-ifosfamide-cisplatin (VIP), vinblastine-ifosfamide-cisplatin (VeIP), or paclitaxel-ifosfamide-cisplatin (TIP). HDCT with a carboplatin-etoposide backbone followed by bone marrow transplant (BMT) was first investigated at Indiana University in 1986. In 1996, BMT was replaced by peripheral-blood stem-cell transplant (PBSCT) allowing for more rapid engraftment and hence fewer delays in delivering a second course of HDCT. With this regimen, cures were achieved in the second-line, third-line, or further setting [3, 4]. To our knowledge, there have been no randomized studies demonstrating superiority of one form of salvage chemotherapy over another (standard-dose vs. HDCT) nor are there studies comparing one HDCT regimen to another.

In a series of 364 consecutive patients with relapsed GCT treated at Indiana University from 2004-2014, HDCT plus PBSCT achieved cures in 60% of patients [5]. 94% of patients received the planned 2 courses of HDCT, each consisting of carboplatin 700 mg/m^2^ plus etoposide 750 mg/m^2^ on days -5,-4, -3 followed by stem-cell infusion on day 0. Remission rates were higher when HDCT was utilized as initial salvage vs. ≥3^rd^ line therapy (63% vs. 49%). Notably, patients with relapsed seminoma had a 90% cure rate. Cures were also achieved in patients with poor prognostic features: platinum refractory defined as progression ≤4 weeks after first-line platinum-based chemotherapy (33%), primary mediastinal non-seminomatous GCT (PMNSGCT) (23%), and progressive brain metastasis at initiation of HDCT (40%). There were 9 treatment-related deaths (2.5%) with 6 occurring ≤30 days from completion of HDCT. Secondary leukemia developed in 5 patients.

The choice of initial salvage chemotherapy for relapsed GCT remains unsettled. In our opinion, patients who experience relapse after initial chemotherapy require multi-disciplinary expertise and should be referred to centers of testicular cancer excellence. Although there are no phase 3 studies demonstrating the superiority of one approach over another, a large multi-national retrospective analysis including 1,594 patients with relapsed GCT was published in 2011 [6]. This study included patients with varied prognostic features and heterogeneous treatment regimens; results showed superior outcomes with HDCT vs. standard-dose chemotherapy (56% decreased risk of progression) translating into an overall survival in all prognostic subgroups except the low-risk category. The difference in cure rates was most pronounced in the very high-risk group: 27% in HDCT vs. 3% in standard-dose chemotherapy. An ongoing multi-national randomized phase 3 trial (TIGER) is investigating HDCT with the TI-CE (paclitaxel+ifosfamide→high-dose carboplatin and etoposidex3) vs. standard-dose chemotherapy with TIPx4 as initial salvage (ClinicalTrials.gov number, NCT02375204).

At Indiana University, our preferred approach is to use tandem HDCT with carboplatin-etoposide plus PBSCT for most patients as second-line chemotherapy. There are special considerations in certain patient populations. (1) For patients who relapse with anatomically confined disease, salvage surgery is our preferred approach. When performed by experienced surgeons, this has potential for cure with significantly less long-term morbidity compared to salvage chemotherapy [2]. (2) Patients with late relapse (>2 years after initial platinum-based chemotherapy) have marginal benefit from salvage standard-dose or HDCT and our preferred approach is surgical resection if feasible. The optimal treatment for patients with multifocal unresectable late relapse remains unknown. (3) Patients with PMNSGCT have inferior outcomes compared to their testicular primary counterparts with higher chance of relapse after first-line and salvage therapy. While initial salvage results in PMNSGCT were disappointing, switching from BMT to PBSCT allowed for more rapid delivery of the second course of HDCT and improved outcomes.^4,5^ (4) The management of patients with progressive brain metastases at the time of relapse is challenging with no uniform treatment recommendations. Options include chemotherapy, stereotactic or whole brain irradiation, surgery, or multimodality approach. Our experience demonstrates that patients with relapsed GCT and progressive brain metastases, including those with prior loco-regional therapy, are curable with HDCT and PBSCT [7]. Whole brain irradiation is seldom used and our preference is for stereotactic therapy, if needed. (5) Relapsed patients with malignant transformation of teratoma have worse prognosis and optimal salvage therapy is not well defined [8]. (6) Relapsed patients with platinum-refractory disease have poor prognosis and our approach is to proceed directly to salvage HDCT without prior standard-dose chemotherapy. With this approach, 33% of patients with relapsed platinum-refractory patients achieve cures [5]. Although the choice of salvage therapy for relapsed GCT is controversial, we believe there are some patients who are only potentially curable by HDCT plus PBSCT. Patients with high risk features: platinum-refractory, PMNSGCT, or progressive brain metastases, should be evaluated at tertiary care centers for expeditious institution of salvage treatment.

Nabil Adra: Division of Hematology & Medical Oncology, Melvin & Bren Simon Cancer Center, Indiana University School of Medicine, Indianapolis, Indiana, USA
